# An Initial Single-Center European Experience with the Gore Thoracic Branch Endoprosthesis

**DOI:** 10.1093/icvts/ivaf309

**Published:** 2025-12-26

**Authors:** Mark Dirven, Guillaume S C Geuzebroek, Foeke J H Nauta, Rozemarijn J van der Vijver, Loes Knaapen, Tychon E A Geeraedts, Sjoerd F M Jenniskens, Robin H Heijmen

**Affiliations:** Department of Vascular Surgery, Radboud University Medical Center, 6525 GA Nijmegen, Netherlands; Department of Cardiothoracic Surgery, Radboud University Medical Center, 6525 GA Nijmegen, Netherlands; Department of Cardiothoracic Surgery, Radboud University Medical Center, 6525 GA Nijmegen, Netherlands; Department of Vascular Surgery, Radboud University Medical Center, 6525 GA Nijmegen, Netherlands; Department of Vascular Surgery, Radboud University Medical Center, 6525 GA Nijmegen, Netherlands; Department of Medical Imaging, Radboud University Medical Center, 6525 GA Nijmegen, Netherlands; Department of Medical Imaging, Radboud University Medical Center, 6525 GA Nijmegen, Netherlands; Department of Cardiothoracic Surgery, Radboud University Medical Center, 6525 GA Nijmegen, Netherlands

**Keywords:** thoracic aortic pathology, single branch, dissection

## Abstract

**Objectives:**

To report an initial experience with a novel off-the-shelf single branched thoracic aortic stent graft preserving various aortic arch vessels.

**Methods:**

Our study is a retrospective cohort analysis of the largest European case series to date. We treated twenty patients for various aortic arch and descending pathology in the year 2024 and 2025.

**Results:**

Twenty patients underwent successful implantation of the thoracic branched endoprosthesis (TBE) in the aortic arch and descending thoracic aorta. The sidebranch was applied to preserve the left subclavian artery in 17 patients, the innominate artery in two and the left carotid artery in one patient. Patients were treated for saccular arch aneurysms, chronic type B dissections with progressive dilatation, type 1a endoleaks after TEVAR, degenerative thoracic aneurysms, localized type A dissections or a first stage Crawford type II thoraco-abdominal aneurysm repair followed by a subsequent visceral branched endoprosthesis. The median follow-up period was six (1-12) months and technical results were satisfying. All TBE stentgrafts were implanted in the desired position with a patent branch on computed tomography angiography (CT-A) scan six weeks postoperatively. There was no in-hospital or 30-day mortality. Unfortunately, two patients suddenly died seven and eight weeks postoperatively of unknown causes. CT-A scan at six weeks showed no abnormalities concerning the aorta or TBE in both patients.

**Conclusions:**

The present study demonstrates satisfying technical results with the GORE TBE which was successfully implanted for multiple indications of aortic arch or descending pathology. Longer follow-up and larger series are needed for verification.

## Graphical abstract

**Figure:**
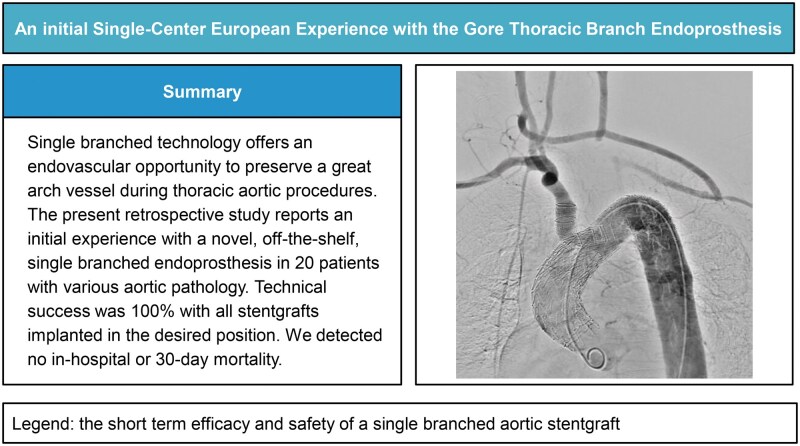

## INTRODUCTION

The introduction of thoracic endovascular aortic repair (TEVAR) has changed clinical practice dramatically. TEVAR is often the first choice of treatment and increasingly extended into the aortic arch and ascending aorta.^1^^,^^2^ The question on whether or not to revascularize the left subclavian artery (LSA) remains a topic of discussion when extending into zone two. LSA coverage is often required to obtain an adequate proximal seal. Possible disadvantages include risk of left arm ischaemia, posterior cerebral stroke and spinal cord ischaemia caused by reduced vertebral artery inflow. Consensus is that patients with a dominant left vertebral artery or a patent internal mammary artery conduit perfusing a coronary vessel, always require revascularization to avoid arm or coronary ischaemia or a posterior cerebral stroke. According to recent European Society for Vascular Surgery guidelines, LSA revascularization should be considered in elective setting.^2^ Bradshaw et al conclude that intentional LSA coverage without revascularization is associated with an increased risk of 30-day stroke and spinal cord ischaemia.^3^ Some studies report a higher incidence of arm ischaemia and recommend standard LSA revascularization.^4^^,^^5^ Others consider LSA coverage safe and unassociated with postoperative morbidity.^6–8^ Techniques such as carotid subclavian bypass or transposition are typically performed prior to TEVAR when LSA preservation is required. Despite good long-term patency, surgical LSA revascularization may cause perioperative complications such as stroke, bleeding, infection, graft occlusion and nerve injury leading to hoarseness, diaphragmic palsy or Horner’s syndrome.^9–12^ Optional full endovascular solutions include custom made TEVAR, in situ fenestration, chimney or snorkel techniques.^13–15^

The Thoracic Branched Endoprosthesis (TBE) was introduced in Europe in the year 2024. The TBE is an FDA/MDR and CE marked stent graft equipped with a single retrograde branch (W. L. Gore & Associates, Inc, Flagstaff, Ariz). The ‘off-the-shelf’ availability provides a new, full endovascular option to preserve the LSA or other great arch vessels in elective or emergent settings. Our report describes a series of twenty patients treated with the TBE for multiple indications. The aim of the present study was to analyze the short-term safety and efficacy of the TBE in various aortic arch and descending pathology.

## PATIENTS AND METHODS

Our study is a retrospective cohort analysis of the largest European case series to date and describes the short-term outcomes of our initial experience with the TBE. Data were collected from patient medical electronic records. We treated twenty patients for various aortic arch and descending pathology in the year 2024 and 2025. Patient demography is described in **Table 1**. Patients who underwent a TBE operation in elective or subacute setting between June 2024 and 2025 were included in our study. Patients were excluded in case of objection to the use of medical data for research purposes. Our primary end-points were technical success and 30-day mortality. Technical success was defined as freedom from type 1 or 3 endoleak on six week postoperative computed tomography angiography (CT-A) scan. Secondary end-points were full aneurysm exclusion, TBE branch patency and neurological outcomes including stroke and paraplegia. Continuous variables are described as medians with interquartile range (IQR, 25th-75th percentile) unless otherwise specified.

**Table 1.** Patient Demography

Characteristics	Value
*Gender*	
Male (n)	10 (50%)
Female (n)	10 (50%)
Age, median (range)	72 (52-81)
Smoking (n)	7
Pulmonary disease (n)	7
Cardiac disease (n)	13
Hypertension (n)	10
Previous CVA/TIA (n)	2
Diabetes (n)	1
Renal dysfunction (n)	6
Active Malignancy (n)	1
Previous Aortic repair (n)	
Zone 2 partial arch	2
TEVAR descending	3
Open descending	1

Preoperatively, all patients received extensive consultation and physical examination by a vascular or cardiothoracic surgeon. Twelve patients used aspirin or clopidogrel which was continued pre and postoperatively. Eight patients used Non vitamin-k antagonist Oral Anti-Coagulants or vitamin-k antagonists. These were temporarily interrupted and restarted postoperatively. All patients were screened by a dedicated anesthesiologist. Adjunctive screening by a cardiologist, pulmonologist or geriatrician was performed if necessary. The indication for TBE implantation was assessed by our multidisciplinary aortic meeting. Patients were selected only if vascular anatomy met the anatomical criteria for implantation and if the operation could be performed under general anaesthesia. Anatomical criteria were a minimum proximal sealing zone of 20 millimeters (mm) in length and a proximal aortic diameter between 24 and 42 mm. An adequate landing zone for the branch target stent was defined as vessel diameter between 6 and 18 mm and a minimum of 15 mm in length. Detailed preoperative planning based on CT-A scan measurements was agreed to by both W.L. Gore representatives and the operating team. Measurements were performed using dedicated software (Aquarius, Tera Recon). Patients were operated on by a dedicated team of two interventional radiologists, two vascular and two cardiothoracic surgeons, all trained and certified by W.L. Gore for TBE implantation. Every operation was performed by one cardiothoracic surgeon, one vascular surgeon and one interventional radiologist. All operations were performed at a hybrid operating theatre with a cardiovascular anesthesiologist. Percutaneous access was applied in the groin (Abbott, Perclose, ProStyle), creating smaller incisions compared to open femoral access. A small surgical incision in the elbow provided was preferred since percutaneous closure devices regularly fail in the brachial artery. ACT-guided heparin dosing of 200 seconds was applied after introduction of access sheaths. Induction of hypotension by placement of an occlusive inferior vena cava balloon was performed during TBE deployment with a target systolic blood pressure of 80 mmHg. This technique ensures a gradual drop in systolic pressure facilitating precise placement in the aortic arch. Blood pressure rises quickly after de-sufflation of the balloon.^16^ Alternative techniques are medicinally induced hypotension, by which blood pressure regulation is less controllable, or rapid ventricular pacing of the heart harboring more risk of cardiac complications.

### TBE device characteristics

**Figure 1.**
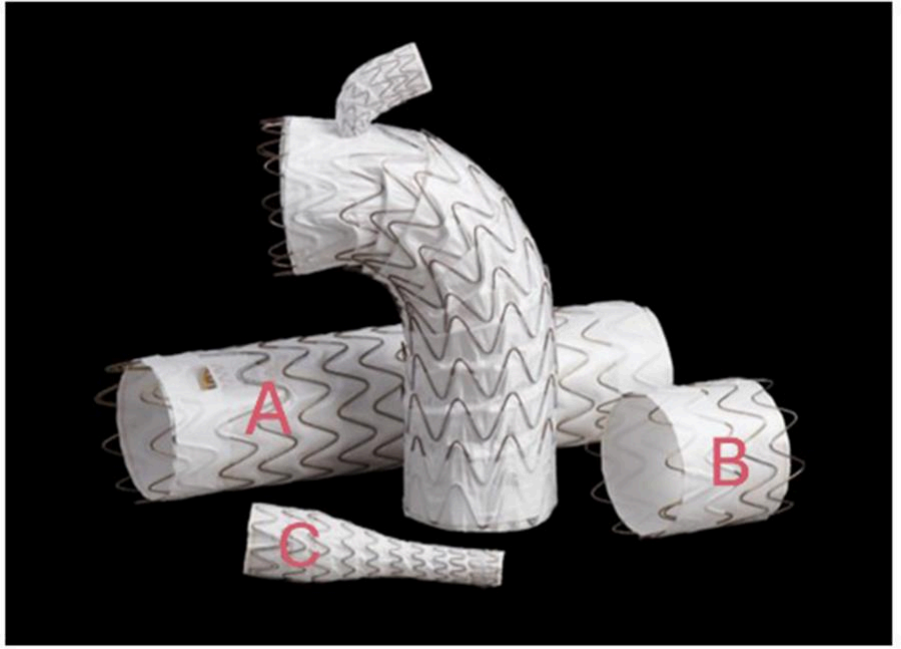
TBE: Three Components (Published with Permission of W.L. Gore and Associates). (A) Main aortic component, (B) Proximal aortic extender, (C) Dedicated target branch stent

The TBE consists of three components (**Figure 1**). The main aortic component (A) varies from 21 to 45 mm in diameter and has a separate side branch portal. The device is available in two configurations: a 40 mm proximal segment with a 12 mm portal and a shorter 20-25 mm proximal segment with an 8 mm portal. Both may be used for various indications dependent on the length of the proximal sealing zone and diameter of the arch vessel. The main component is introduced via femoral access over two wires. One stiff wire to guide it into the aortic arch and one snared wire through the target arch vessel. The second component (B) is a short proximal aortic extender in various diameters compatible to the TBE. The third component (C) is a dedicated target branch stent which is tapered to the size of the chosen portal (8 or 12 mm) and designed to lock in by nitinol anchors. This component is introduced via femoral access over the snared wire into the arch vessel. Dependent on target vessel diameter, distal diameters from 8 to 20 mm are available. Extensive details regarding procedural steps and device characteristics have been described by Patel et al.^17^

### Ethical approval and informed consent

The research protocol of this study was scrutinized by our local Medical Ethics Review Committee and considered not subject to the Medical Research Involving Human Subjects Act. Patients were included only if consent for use of clinical data for research purposes was provided. Consent was obtained via opt-in procedures in patient medical electronic records. The use of medical data for this study adheres to the standard operating procedure of Radboudumc, which outlines the policy for using existing medical data or material from Radboudumc patients. All patients provided informed consent for TBE implantation and analysis of CT-A scans by W.L. Gore.

## RESULTS

**Figure 2.**
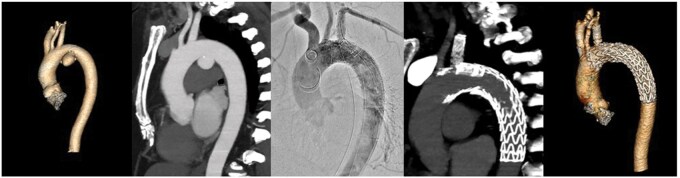
Patient Treated for a Saccular Descending Aortic Aneurysm

**Figure 3.**
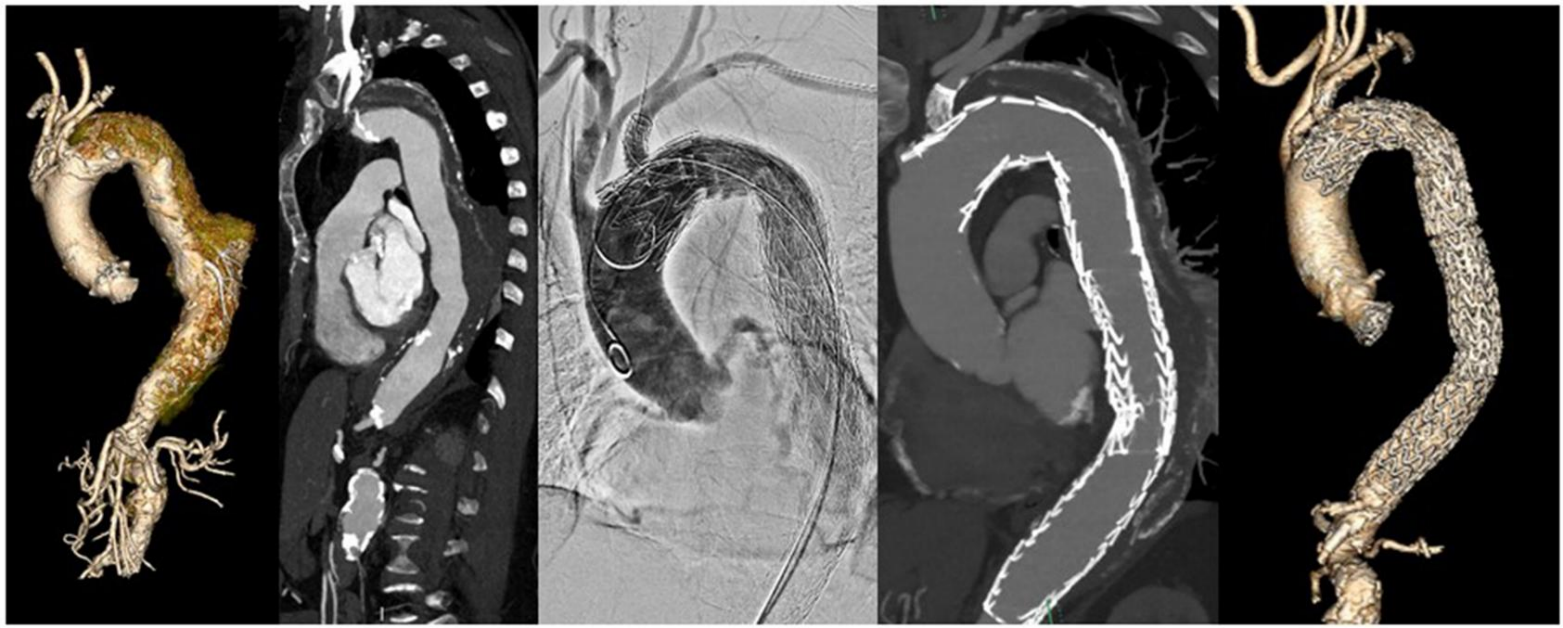
Patient Treated for a Degenerative Descending Aortic Aneurysm

**Figure 4.**
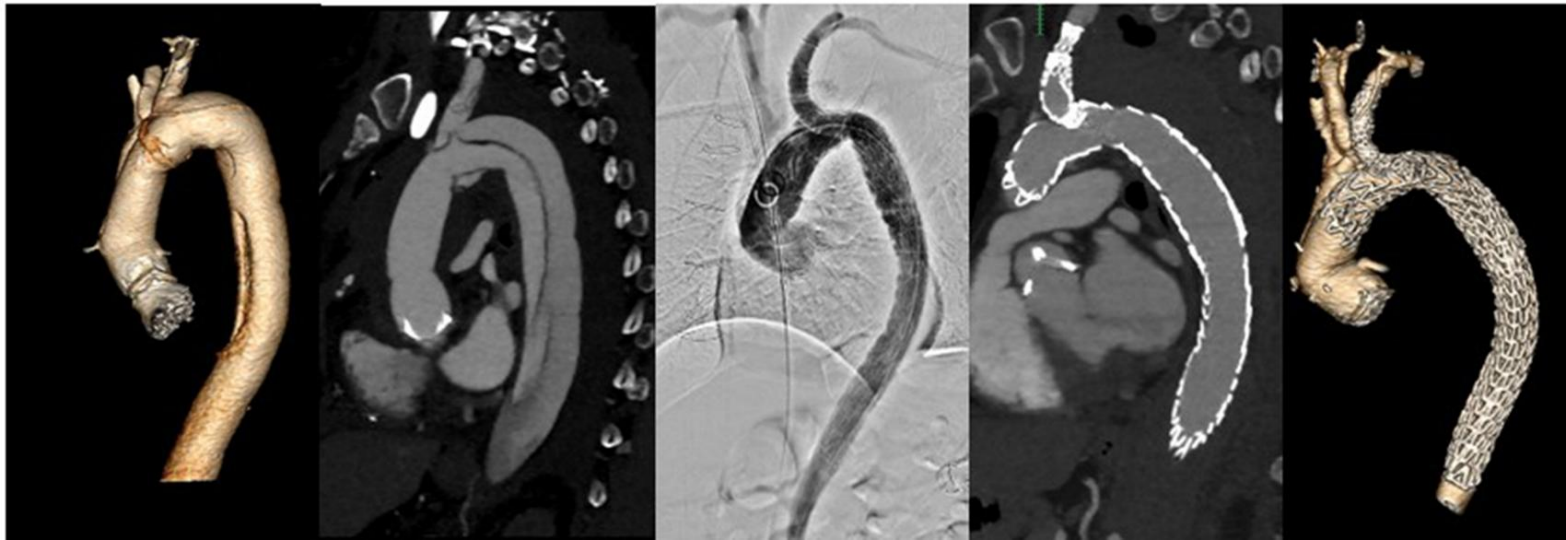
Patient Treated for a Chronic Dissecting Arch and Descending Aneurysm. Central Image/Graphical Abstract. Patient Treated for a Localized Type A Dissection by Carotid-Carotid-Subclavian Bypass and TBE in Zone 0.

Aneurysm morphology and peri- and postprocedural data are described in **Table 2**. Procedural, pre- and postoperative CT-A images are displayed in **Figures 2, 3 and 4**. Our follow-up period was six months (5-10). Patients were followed-up in standard care protocols for TEVAR in the Netherlands. On discharge, patients were extensively informed on possible adverse events and provided with emergent contact data of our hospital. CT-A scans were performed approximately six weeks postoperatively in all patients followed by consultation at our outpatient clinic. Three patients reached a follow-up of one year and underwent a CT-A scan at that time. Both patients treated for a type A dissection were scanned additionally the day after surgery.

**Table 2.** Aneurysm Morphology and Procedural Data

Indication	Patient	Diameter (mm)							Operating time (mins)	Contrast iodine (ml)
		Target Vessel	Aortic maximal	Aortic Neck	Aortic distal	TBE	Side Branch	Target Stent		
Saccular Aortic Aneurysm opposite to the LSA	1	12	50	35	33	40	8	15	123	249
	2	8	46	26	24	31	8	10	103	170
	3	12	53	28	24	31	8	15	92	120
Aneurysmatic Type B dissection	1	11	56	37	26	45	8	15	110	200
	2	10	44	25	25	31	12	15	177	335
	3	12	59	31	32	37	8	15	118	210
	4	10	62	34	28	37	8	12	125	332
	5	12	60	37	40	40	12	17	152	360
Type 1a endoleak after TEVAR/BEVAR	1	12	62	37	40	45	8	15	113	159
	2	8	52	30	31	34	8	10	83	125
	3	10	76	40	26	45	8	12	153	180
Primary degenerative arch or descending (pseudo) aneurysm	1	9	61	37	31	45	8	12	134	175
	2	8	28	23	24	28	8	10	56	100
	3	9	63	28	28	34	8	12	111	200
	4	7	68	22	32	28	8	10	332	196
Localized type A dissection	1	12	50	34	32	37	12	15	225	224
	2	11	33	25	28	31	12	15	116	188
First stage Crawford type 2 TAAA	1	13	60	28	–	34	12	17	152	200
	2	11	60	29	–	34	12	15	78	160
	3	8	67	39	–	45	8	10	100	150

Ten male and female patients, age 72 years (69-75), underwent a successful and uncomplicated TBE implantation in aortic arch zone zero, one or two. Prior to placement we occluded the coeliac trunc with an amplatzer plug in one patient, after stenting a stenosed superior mesenteric artery, to create an adequate distal landing zone. Another patient underwent a left vertebral artery embolization, originating from the proximal sealing zone in the aortic arch, to avoid endoleakage. The circle of Willis was intact in this patient with a patent contralateral vertebral and basilar artery. None of the patients required blood pressure regulation or a spinal drain for treatment of spinal cord ischaemia postoperatively.

The TBE diameter was 37 mm (31-40) with a 15 mm (11-15) diameter of the branch target stent. Distal extension with a second or third stent graft (Gore, C-TAG) was conducted in 12 patients (60%) to obtain full aneurysm exclusion. One patient received an additional TBE cuff for inadequate proximal fixation peroperatively. Operations were performed for various indications. A TBE was implanted in three patients (15%) with a thoraco-abdominal aneurysm in preparation of a branched thoraco-abdominal endoprosthesis. Three patients were treated for saccular arch aneurysms (15%), five patients for a chronic type B dissection (25%), three patients for a type 1a endoleak after TEVAR (15%), four patients for a degenerative thoracic aneurysm (20%) and two for a localized type A dissection (10%). These two patients were referred from other hospitals and considered unfit for open surgery due to extensive comorbidity. The referring physicians requested a minimally invasive endovascular treatment at our center. Both patients were haemodynamically stable with an estimated life expectancy of three years. However, after careful consideration and preoperative work-up, we agreed that open surgery was not an option in either patient. A TBE was implanted in both patients branching the innominate artery after carotid-carotid-subclavian bypass. Seventeen branch stents were implanted in the left subclavian artery. In one patient, the branch stent was placed in the left carotid artery after carotid-subclavian bypass. A Frozen Elephant Trunc and lateral surgical approach with deep hypothermic circulatory arrest were also discussed. However, our multidisciplinary meeting and the patient preferred a minimally invasive option.

### Technical results during our entire follow-up

Operating time was 117 min (102-152). We managed to implant one TBE within the hour in a patient with uncomplicated anatomy. Our most extensive procedure concerned a patient with a severely elongated thoracic aorta, providing significant challenges in correct placement of the branch. Overall periprocedural blood loss was neglectable and 192 milliliters of iodine contrast media (160-218) was used peroperatively. Fluoroscopy time was 27 min (23-35). All devices were positioned as desired on CT-A scan six weeks postoperatively. No stent graft migration or type one of three endoleaks were detected, attributing to a technical success rate of 100%. Full aneurysm exclusion was obtained in 17 patients. Aneurysms were, as anticipated, not excluded in the three patients treated in preparation of a branched thoraco-abdominal endoprosthesis. Adequate patency of the branch stents, without kinking, was observed in all patients. We detected a type three endoleak in one patient treated for a type A dissection the day after surgery on CT-A scan. This was not observed on completion angiography. This endoleak, caused by inadequate sealing of the branch target stent in the innominate artery, was successfully treated with an adjunctive stent resulting in full exclusion of pathology. We detected a type II endoleak in two patients originating from intercostal arteries without aneurysm growth. These patients were treated for a descending and an arch aneurysm. No adjunctive treatment was performed. Three patients treated for a saccular arch aneurysm received a CT-A scan at one year follow-up. Adequate positioning of the TBE, branch patency and full aneurysm exclusion was observed.

### Postoperative complications and mortality

We observed no postoperative nerve damage, haematoma requiring re-exploration, arm or leg ischaemia in relation to access sites in the femoral or brachial arteries. No internal or external bleeding was observed. None of the patients had signs of paraplegia or stroke and no transient ischaemic attacks or paraparesis occurred. Nineteen patients (95%) were discharged the day after surgery and admitted for a total of two days. One patient was admitted four days for a postoperative fever which resolved spontaneously. Another patient was re-admitted 11 days postoperatively with a fever and overall weakness. Blood cultures were negative and Positron Emission Tomography scan revealed no signs of infection. During re-admission the fever resolved and patient was discharged in good clinical condition. Both patients were treated for a chronic type B dissecting aneurysm. There was no in-hospital or 30-day mortality. Unfortunately, one patient died at home seven weeks postoperatively. The TBE was implanted for a rapidly growing type B dissecting aneurysm. The patient was also burdened with a dilated ascending aorta of 53 mm which might have caused a type A dissection or rupture. A routine CT-A scan five days prior to this event, revealed an adequate TBE position and full aneurysm exclusion. Another patient died two months after surgery. The TBE was implanted for a degenerative descending aortic aneurysm. Postoperative CT-A scan at six weeks displayed no abnormalities and full aneurysm exclusion. This patient was admitted at our emergency room in shock rapidly dying thereafter. Echography on site revealed no free fluid in the thoracic or abdominal compartment. Patient was treated for severe coronary stenosis in a referring hospital and might have died from myocardial infarction.

## DISCUSSION

The TBE is a new stent graft on the European market and commercially available since 2024. The device was previously introduced in the United States. Large European series are therefore lacking but several American groups have published results for various elective and emergent indications. These reports describe high technical success percentages and low mortality, stroke and paraplegia rates. Dake reports a freedom from type I or III endoleak of 96.7% and no 30-day mortality in a multicentre, feasibility study. Five patients had died at one year follow-up but causes were not device related.^18^ Liang published a multicentre feasibility trial in 2022 implanting the TBE in 31 patients for aneurysmal disease. Freedom from death at one and three years of 90% and 84% were reported.^19^ Desai published a prospective trial including 132 patients treated for acute, chronic or residual type B dissections. A 30-day stroke and mortality rate of 1.5% and 4.5% was reported with the loss of only one side branch.^20^ The same group performed a multicentre non randomized prospective trial including 238 patients with comparable results.^21^ Dilosa reported an experience in 20 patients with acute blunt thoracic aortic injuries, complicated dissections or ruptured aneurysms. All pathology was excluded successfully and no major complications within 30 days were reported.^22^ The same group recently published a retrospective review including seven sites and 356 patients with acute pathology. Technical success was 99% and the authors concluded that emergent TBE implantation offers low mortality, stroke and paraplegia risk.^23^

A single branched thoracic aortic endoprosthesis has been available in Asia for several years. Jing published a prospective multicentre trial with 73 patients concluding that the Castor device (Microport Medical) is safe and effective in type B aortic dissections. Technical success was 97% with two early branch occlusions. Mortality rates were 5% and 7% at one and six years respectively.^23^ Additional reports sustain these results and suggest that single branched technology offers a compelling substitute for open surgical procedures.^24–27^ A third and much older device is the Inoue single branched stent graft. One report describes less favourable results in 17 patients with multiple re-interventions.^28^

To our knowledge, this is one of the first European series reporting short-term TBE results for various pathology. TBE implantation is, in our opinion, best performed by an experienced multidisciplinary aortic team, equipped to treat thoracic aortic pathology open and endovascularly. The device has a gentle learning curve and allows for precise placement in experienced hands. The TBE is pre-loaded on the Gore TAG platform and therefore not equipped with two-staged, conformable TAG active control technology. The device deploys instantly when released and cannot be repositioned or curved like the conformable TAG. Although we have not observed misplacement or extensive bird beaking, this might be a future point of improvement.

Limitations of the present study are a retrospective design and short-term follow-up. Our technical results and 30-day mortality rate are satisfying and comparable to previous American reports. Full aneurysm exclusion, as intended in 17 patients was achieved. No 30-day mortality, stroke or paraplegia was detected. However, two patients died at seven and eight weeks follow-up of unknown causes. An autopsy was not performed in either patient. Although we cannot directly relate these events to TBE implantation, results must be interpreted with caution considering this is a small and heterogenous cohort. Another point of caution is that many TEVAR complications emerge within longer follow-up times than six weeks to a year. Since our follow-up is short, future studies are needed to verify our results and to compare the TBE and Castor devices. Comparisons to other techniques such as extra-anatomical debranching of arch vessels followed by TEVAR, chimney stenting and in situ fenestration could also be of great value.

Our study demonstrates that the TBE is effectively applicable for a wide range of aortic arch and descending pathology in the elective and subacute setting. Future application could be more aimed at emergent cases or zone zero and one repair as performed in three of our patients.

## CONCLUSION

Our study demonstrated a 100% technical success after TBE implantation in 20 patients. No 30-day mortality, stroke or paraplegia was observed in our follow-up. Larger prospective multicentre studies are needed for verification of these results.

## Data Availability

The data for the analysis of this study is stored on the Radboudumc surgical departments’ shared research drive. All data for this study were anonymously collected and entered into a Microsoft Excel file. The privacy of the participants in this study was warranted by the use of encrypted and unique individual subject codes. By complying with the standard operating procedure ‘Data Management Requirements’ set by the Radboudumc, conditions according to the WMA declaration of Helsinki and Taipei were met.
